# Effectiveness of a 6-Month Nutrition Intervention in People Living with HIV and Prediabetes Progressing through Stages of Change towards Positive Health Behavior

**DOI:** 10.3390/ijerph192214675

**Published:** 2022-11-08

**Authors:** Alicia Sneij, Adriana Campa, Fatma Huffman, Florence George, Mary Jo Trepka, Sabrina Sales Martinez, Marianna Baum

**Affiliations:** 1 Department of Physical Medicine and Rehabilitation, University of Miami Miller School of Medicine, Miami, FL 33136, USA; 2 Department of Dietetics and Nutrition, Florida International University, Miami, FL 33199, USA; 3 Department of Mathematics and Statistics, Florida International University, Miami, FL 33199, USA; 4 Department of Epidemiology, Florida International University, Miami, FL 33199, USA

**Keywords:** prediabetes, HIV, nutrition, intervention, health

## Abstract

The prevalence of prediabetes in people living with human immunodeficiency virus (HIV) is two to three times higher than that of the general population. The aim of this study was to assess the effectiveness of an intervention in guiding low-income people living with HIV (PLWH) and prediabetes through the stages of change and promote self-efficacy of positive health behavior. Methods: A 6- month randomized, controlled intervention was conducted where participants (N = 38) were randomized into the intervention group (n = 20) or the control group (n = 18). The participants’ stages of change, nutrition knowledge, and self-efficacy were assessed using questionnaires. Participants were recruited in August 2017–December 2018, were HIV seropositive, had undetectable viral load, were prediabetic, and not currently receiving glucose-altering medications. Participants randomized into the intervention group received medical nutrition therapy/counseling and nutrition education; participants randomized into the control group received educational material related to nutrition, HIV, and prediabetes at baseline. Primary outcome measures were progression through the stages of change as measured by the transtheoretical (“stages of change”) model, improvements in nutrition knowledge, and self-efficacy of the participants. Results: Significant improvement in stage of behavioral change was observed in the intervention group for physical activity, fruit/vegetable intake, fiber intake as well as nutrition knowledge and self-efficacy; however, no significant changes were observed in the control group. Conclusions: A nutrition intervention was effective in promoting positive health behavior by progressing participants through the stages of behavioral change in low-income people living with HIV and prediabetes.

## 1. Introduction

### 1.1. Study Population

The prevalence of prediabetes in people living with HIV (PLWH) is two to three times higher than that of the general population [1,2,3]. The increased risk for prediabetes in this population is multifactorial, including but not limited to HIV infection, chronic use of antiretroviral therapy (ART), and aging due to the success of the ART treatment [4,5,6,7,8]. As diabetes and prediabetes are known to be delayed with healthy-eating and lifestyle habits [9,10], it is important to develop effective behavioral interventions to promote healthy changes to balance the higher risk for prediabetes in PLWH.

Many PLWH tend to be low income and of lower socioeconomic status, with greater prevalence of HIV infection occurring at the lower end of income and socioeconomic status [11]. The additional factors of low income and socioeconomic status may contribute to the difficulty in implementing effective nutrition interventions in this unique population compared to other population groups. 

### 1.2. Behavioral Change Models

Behavioral change frameworks have proven effective in creating positive behavioral change, including but not limited to the health belief model [12,13] and the transtheoretical model [14,15]. The health belief model was developed to help understand and explain health-related behavior based on perceived barriers, benefits, self-efficacy, and threat. Based on these parameters, an individual’s behavior may be effectively altered if any of these domains are properly addressed [12,13,14]. The literature suggests that based on the health belief model, people need to perceive both risk of disease and potential benefit of implementing a behavior change [16]. As people with prediabetes have increased risk of developing diabetes [17,18], they are perfect candidates for implementing an intervention based on the health belief model [16].

Another behavioral change model is the transtheoretical model. The transtheoretical model is an integrative behavioral change model that assesses an individual’s readiness to make certain positive changes and is composed of constructs such as stages of change and self-efficacy [14,15,16,19]. The transtheoretical model proposes that for effective behavior change to take place, an individual must progress through five stages of change: precontemplation, contemplation, preparation, action, and maintenance [19].

### 1.3. Nutrition Interventions

Although studies incorporating proven behavioral change models have been conducted in the general population [20,21,22], research utilizing behavioral change models and assessing the different constructs is limited in PLWH, a unique population group with high risk for diabetes [1,2,3,4,5,6,7]. Therefore, the objective of this study was to assess the effectiveness of a 6-month lifestyle/nutrition intervention in progressing low-income people living with HIV and prediabetes through the stages of change towards positive health behavior and promote self-efficacy, as measured by the domains and instruments of the health belief model and transtheoretical (“stages of change”) model. The authors of this manuscript hypothesized that a 6-month lifestyle/nutrition intervention will be effective in transitioning low-income people living with HIV and prediabetes through the stages of change towards positive health behavior and promote self-efficacy. Results related to diabetes risk reduction in this sample population were previously published [23].

## 2. Material and Methods

### 2.1. Study Design and Recruitment

The study protocol was approved by the Florida International University (FIU) Institutional Review Board (IRB-17-0058-CR01) prior to the start of the study. Data collection started in August 2017 and concluded in December 2018. Participants were recruited from the Miami Adult Studies on HIV (MASH) cohort [24]. Eligibility criteria for the MASH cohort are HIV seropositive, 18–65 years of age, receiving stable ART for at least 6 months and having undetectable HIV viral load (<50 copies/mL). The MASH cohort consists of mostly African Americans (70%), Hispanics (18%) and others (6%), which are self-reported [24]. MASH cohort participants provided consent to review their medical documentation for eligibility and to participate in the study procedures, which were explained to them in detail. Written informed consent was provided by each participant prior to participation in the study. 

Inclusion criteria for the current study were HIV seropositive, prediabetic, 18–65 years of age, receiving stable ART for at least 6 months, undetectable HIV viral load (<50 copies/mL) and English speaking. Participants were excluded if they did not meet the inclusion criteria and if they had any previous history of type 2 diabetes, concomitant use of glucose-altering medication (corticosteroids, etc.), use of weight-loss drugs, pregnancy or breastfeeding and/or refusal or inability to give informed consent to participate in the study.

Baseline population characteristics for birth sex, age and annual income were self-reported, and variables including body mass index, waist circumference, hip circumference and waist-to-hip ratio were measured/calculated at the research clinic. Blood was drawn (<30 mL) at baseline and at the 6-month follow-up. Participants were determined to be “prediabetic” based on the American Diabetes Association diagnostic criteria of fasting blood glucose of 100 mg/dL to 125 mg/dL (fasting for ≥8 h) [25].

**Figure 1:**
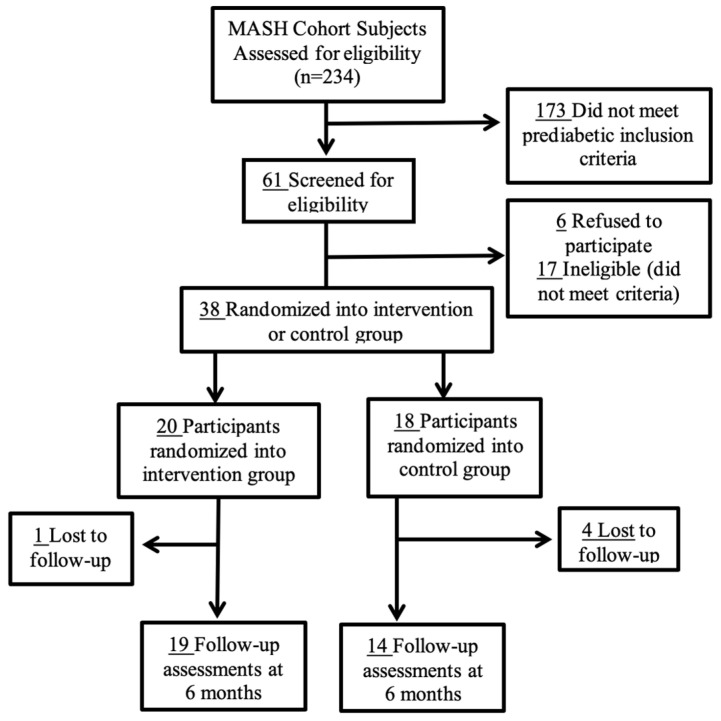
CONSORT Diagram.

From the MASH cohort, 234 participants were assessed for eligibility, of which 190 participants did not meet the inclusion criteria, and six participants refused to participate in the current study (Figure 1). Of the 61 participants that met the HIV and prediabetes eligibility, six participants refused to participate and 17 were excluded due to exclusion criteria; five participants were excluded from the study because they were previously initiated on metformin, which is becoming the standard of care for this population. 

Figure 1 illustrates the CONSORT diagram of people living with HIV and prediabetes recruited for a 6-month nutrition intervention to progress through stages of change for health-related parameters.

All participants visited the FIU Research Clinic at the Borinquen Health Care Centers located in Miami, Florida at baseline for assessment and at the 6-month follow-up. Participants were enrolled and randomized by AS into the treatment group (intervention) or the control group. AC generated the random number sequence using a computer random number generator; even numbers were assigned to the intervention group, and odd numbers were assigned to the control. 

Participants were randomized into either the intervention group or the control group in a parallel study design. Participants randomized into the intervention group received individualized sessions with a registered dietitian monthly for 6 months. During the sessions, the dietitian provided medical nutrition therapy that consisted of nutritional diagnosis, therapy, and counseling services. In addition, nutrition education based on the American Diabetes Association Standards of Medical Care was provided to each participant [25]. Each session lasted for approximately an hour. Participants randomized into the control group were given printed educational material related to nutrition, HIV, and prediabetes at baseline. Participants were compensated $5 for each visit to cover their transportation costs.

### 2.2. Intervention

The intervention utilized a curriculum that was specifically tailored to PLWH and provided structure to the intervention; however, each session was individualized and catered to each participant’s nutritional needs and current stage of change at the time of the session. The transtheoretical model and the health belief model are not interventions, but models of behavioral change, and are used to design interventions that promote effective behavioral change. In this current study, the transtheoretical model was used to assess each participant’s stage along the spectrum of readiness to make and maintain certain behavioral change. This knowledge was then used to tailor the intervention specifically to an individual’s needs according to the various stages.

The educational portion of the intervention included the following topics: (1) HIV, ART, and prediabetes—How are they all interrelated? (2) Understanding BMI and the importance of maintaining normal body weight, (3) Dietary intake and the importance of fruits and vegetables, (4) Energy expenditure and the importance of physical activity, (5) Dietary fat and ways to avoid excessive intake, and (6) Alcohol and its effect on the body. In addition to education and counseling, each participant in the intervention group received a small bag of healthy food such as fruit and whole-grain cereal to serve as samples. 

### 2.3. Questionnaires and Assessment Tools

Validated questionnaires were administered at baseline and at the 6-month follow-up visit using an interview-style method. The questionnaires that were administered to the participants in both groups included the Stage of Change Assessment Tool [26], Nutrition Knowledge Assessment Tool [27], and Self-Efficacy Assessment Tool [27,28]. Each assessment tool has been well-validated but tailored to be more applicable to this unique population of interest. Due to the low socioeconomic status of this study population, questions related to gym memberships and attendance were removed, and questions related to fast-food consumption were added to the validated questionnaires. The average annual income of the study population was $13,325 ± $11,495/year and as a result, most participants were not able to afford gym memberships and frequented fast-food chains.

#### 2.3.1. Stage of Change Tool 

The Stage of Change Assessment Tool is based on the transtheoretical model [19,29], which as aforementioned, is a proven behavioral change model that proposes effective behavior change progressing through a series of stages. The Stage of Change Assessment Tool evaluates each participant’s stage of change according to their willingness to make positive changes in their behavior in relation to physical activity, fruit and vegetable intake, fiber intake, fat consumption and usual alcohol consumption. Based on each participant’s answers, they were categorized into the (1) Precontemplation, (2) Contemplation, (3) Preparation, (4) Action or (5) Maintenance Stage. 

**Figure 2:**
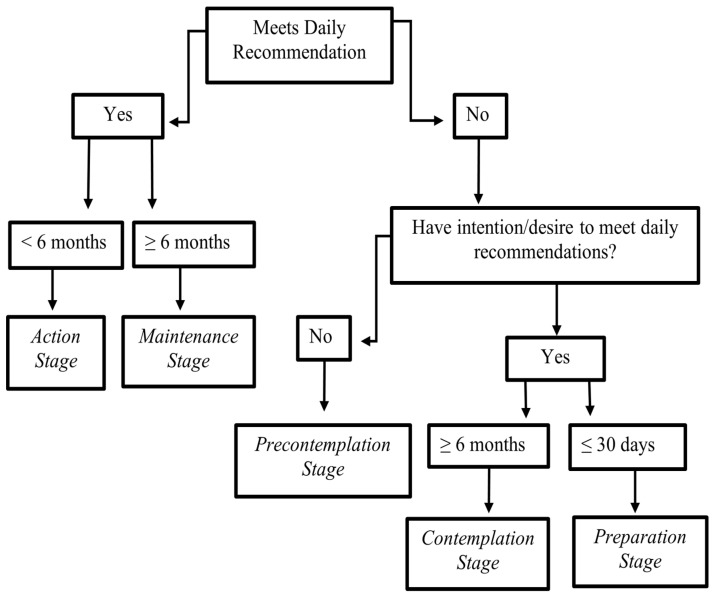
Stage of Change Assessment Flowchart.

The participants’ diets were assessed using the guidelines described in the United States Department of Agriculture 2015 Dietary Guidelines for Americans [30] for each of the assessment components. The stage of change to which each participant was initially assigned was based on their answers to a series of questions in a Stage of Change Assessment Flow Chart (Figure 2).

Figure 2 illustrates the flowchart of stage of change assessment in people living with HIV and prediabetes participating in a 6-month nutrition/lifestyle intervention to progress participants through the stages of change of the transtheoretical model.

If the participants were not currently meeting the recommendations based on the Dietary Guidelines for Americans [30] (Table 1), they were asked if they had any desire to make changes to meet the recommended guidelines. If they were not interested and expressed no desire to make any changes, they were categorized into the Precontemplation Stage of Change. If they expressed that they desired to make changes, they were asked if they were willing to make changes soon (within 30 days), and those stating such willingness were assigned to the Preparation Stage of Change, whereas those who considered making changes in the distant future (≥6 months) were categorized as being in the Contemplation Stage of Change (Figure 2).

If the participants were currently meeting the guidelines at the time of baseline visit, they were asked if they had met the guidelines for at least 6 months, which would put them in the Maintenance Stage of Change. If they had been meeting the guidelines for less than 6 months, then they were categorized in the Action Stage of Change. The stage of change for each participant was assessed at baseline, before being randomized into either study group, and at the end of the study. Participants progressing from an inactive stage of change (precontemplation and contemplation) to an active stage of change (preparation, action, and maintenance) were expected to yield clinical significance [21].

#### 2.3.2. Nutrition Knowledge Assessment Tool

The Nutrition Knowledge Assessment Tool [27] is a true/false and multiple-choice questionnaire with a total of 25 questions. The questionnaire contains a broad spectrum of questions about health and nutrition, including the selection of good sources of fiber and basic understanding of the role of food/nutrition in lowering disease risk. In addition, based on the health belief model, it contained questions to assess the participants’ awareness of the role of nutrition and physical activity in health promotion and disease prevention. Visual aids were used to facilitate the process of assessing nutrition knowledge. Improvement in scores of 2–3 points have been shown to yield statistical and clinical significance [31,32].

#### 2.3.3. Self-Efficacy Tool

Lastly, the Self-Efficacy Assessment Tool [27,28] was used to assess the self-efficacy and confidence of the participants in making certain changes in their lives to promote positive health outcomes. The questionnaire was slightly modified to be more applicable to this study population. Such modifications included questions about fast-food consumption, which is common in this low-income population, and excluded numerous questions about gym memberships and attendance, which are not very common in this low-income study population. 

Areas related to self-efficacy in the questionnaire included confidence in meeting guidelines for fruit and vegetable consumption, physical activity, and meeting health goals. Participants could answer each question in the 25-unit questionnaire using a scale ranging between 0–3 (0 being least confident and 3 being very confident) that reflected their confidence in making decisions regarding their health and lifestyle choices, for a maximum score of 75. Improvements in self-efficacy scores of approximately 5 points have been shown to yield statistical and clinical significance [32].

### 2.4. Statistical Analyses

Data were tested for normality (Shapiro–Wilk test); means/standard deviations were used to describe normally distributed data and frequencies/proportions were used to present categorical data. Parametric tests were used to analyze normally distributed data (paired *t*-test, independent student *t*-test, etc.) and Mann–Whitney U test was used to compare differences in variables that were not normally distributed. Categorical data were presented as frequencies and proportions and analyzed with chi-squared, McNemar’s test, and Fisher’s exact test. Significance was set at *p* ≤ 0.05, and all statistical tests were performed using Statistical Package for the Social Sciences (SPSS) version 23 [33].

## 3. Results 

Participants (N = 38) were randomized into either the intervention group (n = 20) or the control group (n = 18). A few participants (intervention group: n = 1; control group: n = 4) were lost to follow-up and did not complete the study intervention; however, the data were analyzed with intent-to-treat. Baseline characteristics of the study sample can be found in Table 2 for birth sex, age, annual income, body mass index, waist circumference, hip circumference, and waist-to-hip ratio. No significant differences were observed between the intervention group and the control group for any of the baseline parameters presented (Table 2). The study sample consisted of mostly African Americans/Non-Hispanics (68%), White Hispanics (19%), White/Non-Hispanics (8%), and Others (5%), which were self-reported.

To analyze changes in the stage of change of the participants in both the intervention and control groups, the five stages of change were separated into two groups based on a validated staging measure: (a) stages of inaction and (b) stages of action [21]. The stages of inaction included the Precontemplation, Contemplation and Preparation stages of change; the stages of action included the Action and Maintenance stages of change [21].

McNemar’s test was used to assess significant changes in the frequencies of the stages of change in the participants within the study groups. No significant differences were observed in the reported stages of change at baseline between the intervention and control groups (Table 3). However, significant differences were observed in stages of change for physical activity, fruit/vegetable intake and fiber intake when comparing the 6-month values of the intervention group to those of the control group (Table 3). Moreover, a significant difference was observed for fruit/vegetable intake in the intervention group when comparing the pre/post frequencies (*p* = 0.011). No significant differences in frequency changes for pre/post values were observed in the control group (Table 3).

After the 6-month nutrition intervention, significant differences (Fisher’s exact test) were observed in multiple parameters including physical activity, fruit and vegetable intake, and fiber intake (Table 3); significant differences were also observed for nutrition knowledge and self-efficacy (Table 4). No significant differences were reported in fat intake and alcohol consumption between the two study groups at the 6-month follow-up (Table 3). However, a significant difference was observed in fruit/vegetable intake and a tendency to significance in fiber intake when comparing pre/post values in the intervention group (Table 3). Table 4 shows significant differences in nutrition knowledge within the intervention group, when the baseline values were compared to those after 6 months of the intervention. No significant differences were observed in any of the parameters within the control group when comparing the pre/post values (see Table 3 and Table 4).

Up to 70% of the participants in the intervention group (n = 14) and up to 60% of the participants in the control group (n = 11) started at baseline around Preparation and Action Stages of Change (Table 5); however, the participants in the intervention group continued to consistently progress towards the Action Stage of Change, while the participants in the control group remained the same or even regressed towards the Contemplation Stage of Change, as was the case with Fruit and Vegetable Intake (Table 5). 

## 4. Discussion

### 4.1. Progression of Stages of Change

The results from this randomized, controlled clinical trial, based on the health belief model and the transtheoretical model, suggest that a 6-month nutrition/lifestyle intervention is effective in progressing PLWH through the different stages of change. To our knowledge, this is the first study to assess the efficacy of a nutrition intervention in progressing people living with HIV and prediabetes through the stages of change in parameters related to diet and physical activity. 

Similar studies have been performed in other study populations. McKee and colleagues [22] assessed the progression of stages of change in relation to diet and exercise parameters in cardiac rehabilitation patients; however, it was not a randomized, controlled study, which is the gold standard of experimental research. In addition, Greene and colleagues [21] assessed the transition of the stages of change in reducing dietary fat intake in an 18-month dietary feedback intervention in participants consuming >30% of calories from fat per day. However, this study was conducted in the general population and not in PLWH; moreover, it only targeted dietary fat intake, whereas our current study had additional parameters, including physical activity. 

In our study, significant differences were observed between the two study groups at the 6-month follow-up in the following parameters: physical activity, fruit and vegetable intake, fiber intake, nutrition knowledge and self-efficacy. In addition, a significant difference was observed in the pre/post intervention values for fruit and vegetable intake within the intervention group. These findings are supported by McKee and colleagues [22], where significant improvements in stages of change were seen in cardiac rehabilitation patients after 6–8 weeks of intervention in both diet and exercise parameters; however, no detail was provided on the breakdown of diet components, such as fruit/vegetable intake or fiber intake. 

Moreover, our intervention study demonstrated the effectiveness of progressing low-income people living with HIV and prediabetes through the stages of change in increasing daily physical activity, while the participants in the control group did not significantly progress through the stages of change. A study conducted by Vancampfort et al. [34] assessed the proportion of low-income PLWH (N = 75) in the five stages of change with regard to physical activity and found that 29% were in the inactive stages (precontemplation/contemplation/preparation), 55% were in the action stage of change, and 16% were in the maintenance stage of change. These findings are consistent with the results in our study, with 37% of our participants in the inactive stages of change and 63% in the active stages of change (action and maintenance). Although this study by Vancampfort and colleagues [34] assessed the stage of change for physical activity in low-income PLWH, it did not conduct an intervention to promote progression through stages of change towards positive health outcomes as was implemented in this current study. 

Although the participants in this current study were willing to increase intake of fruits and vegetables, they were less inclined to change from refined grains to whole grains to increase fiber intake. Weiss and colleagues [35] demonstrated a greater intake of refined grains compared to whole grains in PLWH. The reluctance of our participants to change from refined grains to whole grains may be due to the unfamiliarity with whole grains in their current diet. The reason whole grains were not part of their current diet is unknown but may be culturally related. To increase fruit and vegetable intake, the participants were encouraged to increase intake of their favorite fruits and vegetables, which they were already familiar with. This may explain why a significant improvement in fiber intake stage of change was observed in the intervention group but not in the control group after the 6-month nutrition intervention. 

Lastly, most of the participants reported not exceeding the daily recommendations for alcohol consumption, with most participants reporting meeting alcohol consumption guidelines in both groups (Table 5); however, there were a select few (n = 4) that were exceeding the recommendations and clearly expressed no desire to change. According to Galvan et al. [36], alcohol consumption is common among PLWH, with rates of heavy drinking approximately twice those found in the general population. It is evident that progressing through stages of change in alcohol consumption was the most difficult decision compared to other behavioral parameters. 

The findings of our intervention study are supported by a study conducted by Duncan et al. [37] that assessed the effectiveness of a 6-month nutrition/physical activity intervention in PLWH. While this cross-over intervention study by Duncan et al. [37] showed the effectiveness of a nutrition/lifestyle intervention in lowering diabetes risk and interviewed the participants to assess the barriers they face in making positive health behavior changes, it did not assess the progression of the stages of change of these participants, which is unique to this study. 

### 4.2. Nutrition Knowledge and Self-Efficacy

The participants in the intervention group significantly improved their nutrition knowledge and self-efficacy scores on the respective assessment tools. Bello et al. [38] found that there is a lack of nutrition knowledge related to healthy meal planning in PLWH with limited resources. Moreover, Muthamia et al. [39] found a significant relationship between nutrition knowledge and various dietary behaviors, such as frequency of fruit/vegetable intake in low-income PLWH. The researchers concluded that nutrition knowledge positively influences the dietary behavior of low-income PLWH, and that nutrition education should include teaching PLWH how to select affordable and nutritious foods. 

These findings are consistent with the findings in our current study, as we observed a significant improvement in nutrition knowledge, along with a significant increase in fruit/vegetable intake, in the intervention group after the 6-month intervention; however, these findings were not observed in the control group. Our intervention study was effective in significantly increasing nutrition knowledge, as well as increasing positive dietary behaviors such as fruit and vegetable intake, in low-income PLWH. 

Furthermore, a significant improvement in self-efficacy was observed in the intervention group compared to the control group. This demonstrates that along with improvement in nutrition knowledge and understanding the link between nutrition and health, based on the health belief model, the participants in the intervention group expressed greater confidence in making changes to promote positive health outcomes compared to the control group. A cross-sectional study conducted by Kelly and colleagues [40] found that self-efficacy was positively associated with meeting the recommendation guidelines for fruit and vegetable intake among PLWH. They concluded that interventions should target self-efficacy to effectively increase fruit/vegetable intake in PLWH and address the perceived benefits of meeting guidelines. These findings are consistent with the findings in our study, as we observed a significant improvement in self-efficacy, as well as in fruit/vegetable intake, in the intervention group, but not in the control group. 

### 4.3. Study Strengths and Limitations 

Strengths of this intervention study include the randomized, controlled clinical trial design, which is considered the gold standard for experimental research, the duration of the intervention (6 months), and the use of proven effective behavioral change models, such as the health belief model and the transtheoretical model. Limitations of the study include the small sample size (N = 38), unblinded study design, and the reliance on self-reported data. Although the questionnaires were an adaptation of validated questionnaires, the adapted form was not validated. Moreover, while a 6-month intervention is sufficient time to observe short-term behavioral change, more studies are needed to assess the long-term effects of the intervention.

## 5. Conclusions

The results from this study suggest that a 6-month intervention was effective in advancing the participants through the stages of change based on the transtheoretical model. While the 6-month lifestyle intervention was able to effectively change behavior in the short-term, more research is needed to confirm if the effects are maintained after the intervention is completed. In view of the results of this study, regular visits with a registered dietitian or another healthcare professional are recommended to provide consistent feedback and maintain or increase motivation among people living with HIV and prediabetes to achieve long-lasting results with health-promoting habits. 

## Figures and Tables

**Table 1:** Key Recommendations from the 2015 Dietary Guidelines for Americans.

Behavior	Guideline/Recommendation
Physical Activity	≥30 min of purposeful physical activity regularly, preferably daily
Fruit and Vegetable Intake	≥2 servings of fruits and ≥3 servings of vegetables per day
Dietary Fiber Intake	≥6 servings of grain products, with ≥3 being whole grains
Dietary Fat Intake	<30% of energy intake from total fat
Alcohol Consumption	≤1 alcoholic beverage per day (women) ≤2 alcoholic beverages per day (men)

Table 1 contains the recommendations from the 2015 Dietary Guidelines for Americans used to determine if people living with HIV and prediabetes in a randomized, controlled clinical trial of a nutrition intervention were meeting recommendations and assess their stage of change at baseline and end-of-study.

**Table 2:** Study Sample Baseline Characteristics of People Living with HIV and Prediabetes Participating in a 6-Month Nutrition Intervention to Progress through Stages of Change for Health-Related Parameters.

Baseline Characteristic	Mean ± SD (N = 38)	Intervention Group (n = 20) Mean ± SD	Control Group (n = 18) Mean ± SD	*p* Value
Birth Sex *	Female: n = 15 (39%) Male: n = 23 ** (61%)	Female: n = 7 (35%) Male: n = 13 (65%)	Female: n = 8 (44%) Male: n = 10 ** (56%)	0.55 ***
Age (years)	56.70 ± 7.40	55.55 ± 6.07	58.00 ± 8.72	0.40
Annual Income (USD/year)	$13,325.03 ± $11,495.00	$13,072.21 ± $8577.63	$13,605.94 ± $14,323.91	0.51
Body Mass Index (kg/m^2^)	30.10 ± 5.50	29.44 ± 5.55	30.74 ± 5.50	0.40
Waist Circumference (inches)	40 ± 4.70	Female: 40.46 ± 6.30	Female: 40.59 ± 5.17	Female: 0.73
		Male: 39.60 ± 3.86	Male: 39.28 ± 4.85	Male: 0.80
		Total: 39.90 ± 4.72	Total: 40.07 ± 4.80	Total: 0.97
Hip Circumference (inches)	42 ± 4.70	Female: 44.53 ± 6.00	Female:43.81 ± 5.20	Female: 0.56
		Male: 41.14 ± 3.76	Male: 41.09 ± 4.46	Male:0.89
		Total: 42.32 ± 4.80	Total: 42.41 ± 4.73	Total: 0.84
Waist-to-hip ratio	0.95 ± 0.06	Female: 0.91 ± 0.05	Female:0.93 ± 0.06	Female: 0.27
		Male: 0.96 ± 0.06	Male:0.96 ± 0.05	Male: 0.80
		Total: 0.94 ± 0.06	Total: 0.95 ± 0.06	Total: 0.84

* Birth Sex reported as percentage female/male. ** One male identified as transgender female, although was not taking hormones. *** Chi-squared statistic: X^2^ = 0.3737. Shapiro–Wilk test of data normality *p*-values: body mass index (*p* = 0.403), waist circumference (*p* = 0.704), hip circumference (*p* = 0.670), waist-to-hip ratio (*p* = 0.200).

**Table 3:** Frequency of Grouped Stages of Change at Baseline and 6 Months and Comparison of Parameter Changes of People Living with HIV and Prediabetes in Intervention and Control Groups Participating in a Nutrition Intervention to Progress through Stages of Change.

Stages of Change	Physical Activity	Fruits and Vegetables	Fiber	Fat	Alcohol
	Baseline/6-Month				
	Intervention Group (n = 20/n = 19)				
Inactive Stages of Change	5/1	13/5	9/4	8/5	6/3
Active Stages of Change	15/18	7/14	11/15	12/14	14/16
McNemar’s Test Pre/Post Comparison (*p*-value)	0.110	0.011 *	0.063	0.227	0.250
	Control Group (n = 18/n = 14)				
Inactive Stages of Change	9/6	11/11	8/8	10/8	4/2
Active Stages of Change	9/8	7/3	10/6	8/6	14/12
McNemar’s Test Pre/Post Comparison (*p*-value)	0.344	0.188	0.313	0.500	0.500
Fisher’s Exact Test Comparison					
Baseline	0.184	0.535	0.615	0.258	0.360
6-Month	0.007 *	0.002 *	0.033 *	0.074	0.649

Table 3 represents the frequencies of inactive (precontemplation, contemplation or preparation) or active (action or maintenance) stages of change of the participants in each study arm at baseline and 6-month follow-up visits. McNemar’s test was used to assess pre/post changes in frequencies of the inactive or active stages of change groups for each parameter (physical activity, fruits/vegetables, fiber, fat, and alcohol intakes). Fisher’s exact test was used to compare the frequencies of inactive and active stages of change groups for each parameter between each study arm at baseline and 6-month follow-up. * Significant differences observed; significance set at *p* ≤ 0.05; one-sided *p*-values presented.

**Table 4:** Comparison of Nutrition Knowledge and Self-Efficacy at Baseline/6 Months and Changes in Intervention and Control Groups of People Living with HIV and Prediabetes Participating in a Nutrition Intervention to Improve Nutrition Knowledge and Self-efficacy.

Parameter	Intervention (Mean ± SD)		Pre/ Post (*p*-Value)	Control (Mean ± SD)		Pre/ Post (*p*-Value)	Comparison Between Groups (*p*-Value)		Parameter Changes Comparison (*p*-Value)
	Baseline	6-Month		Baseline	6-Month		Baseline	6-Month	
Nutrition knowledge score (0–25)	17.74 ± 2.3	20.47 ± 2.5	0.001 *	17.64 ± 2.5	17.64 ± 2.7	0.99	0.60	0.003 *	0.006 *
Nutrition knowledge (%)	70.95 ± 9.0	81.89 ± 9.8	0.001 *	70.57 ± 10.1	67.21 ± 16.1	0.29	0.60	0.003 *	0.002 *
Self-efficacy score (0–75)	63.32 ± 10.21	67.37 ± 6.3	0.000 *	59.50 ± 9.2	61.43 ± 5.2	0.36	0.49	0.007 *	0.014 *
Self-efficacy percentage (%)	83.95 ± 13.8	89.79 ± 8.5	0.000 *	79.36 ± 12.3	82.07 ± 6.8	0.33	0.55	0.009 *	0.014 *

Table 4 shows the (a) average number of questions on the Nutrition Knowledge Assessment Tool (total of 25 questions) that were answered correctly, presented as mean ± SD, (b) the average percentage of correct answers on the Nutrition Knowledge Assessment Tool (max score: 100%), presented as mean ± SD, (c) the average score on the Self-Efficacy Assessment Tool (total of 75 points), presented as mean ± SD, (d) the average percentage of points on the Self-Efficacy Assessment Tool (max score: 100%), presented as mean ± SD. * Significant differences observed; significance set at *p* ≤ 0.05.

**Table 5:** Frequency of Stages of Change for Each Parameter at Baseline and 6 Months for People Living with HIV and Prediabetes in Intervention and Control Groups of a Nutrition Intervention to Progress through Stages of Change.

Stages of Change	Physical Activity	Fruits and Vegetables	Fiber	Fat	Alcohol
	Baseline/6 Months				
	Intervention Group (n = 20/n = 19)				
Precontemplation	1/0	1/0	3/1	1/1	3/1
Contemplation	1/0	1/0	0/0	0/0	1/1
Preparation	3/1	11/5	6/3	7/4	2/1
Action	4/1	3/3	5/3	3/3	1/1
Maintenance	11/17	4/11	6/12	9/11	13/15
	Control Group (n = 18/n = 14)				
Precontemplation	3/2	2/3	0/2	1/3	1/1
Contemplation	0/0	0/1	0/1	0/1	0/0
Preparation	6/4	9/7	8/5	9/4	3/1
Action	2/0	2/0	1/0	1/1	2/2
Maintenance	7/8	5/3	9/6	7/5	12/10
